# Activation of signaling pathways in models of t(6;9)-acute myeloid leukemia

**DOI:** 10.1007/s00277-022-04905-9

**Published:** 2022-08-08

**Authors:** Claudia Chiriches, Dilawar Khan, Maria Wieske, Nathalie Guillen, Michal Rokicki, Carol Guy, Marieangela Wilson, Kate J. Heesom, Oliver Gerhard Ottmann, Martin Ruthardt

**Affiliations:** 1 Division of Cancer and Genetics, Department of Haematology, School of Medicine, Cardiff University, Cardiff, CF14 4XN UK; 2 Experimental Clinical Medical Center (ECMC) Cardiff, School of Medicine, Cardiff University, Cardiff, CF14 4XN UK; 3 Department of Hematology, J.W. Goethe University, Theodor-Stern-Kai 7, 60590 Frankfurt, Germany; 4 Biomedical Sciences Building, University of Bristol Proteomics Facility, Bristol, BS8 1TD UK

**Keywords:** AML, Therapy resistance, t(6, 9), DEK/CAN, ETV6/ABL1, Signaling pathways

## Abstract

Patients within the WHO-subgroup of t(6;9)-positive acute myeloid leukemia (AML) differ from other AML subgroups as they are characterised by younger age and a grim prognosis. Leukemic transformation can often be attributed to single chromosomal aberrations encoding oncogenes, in the case of t(6;9)-AML to the fusion protein DEK-CAN (also called DEK-NUP214). As being a rare disease there is the urgent need for models of t(6;9)-AML. The only cell line derived from a t(6;9)-AML patient currently available is FKH1. By using phospho-proteomics on FKH1 cells, we found a strongly activated ABL1 kinase. Further investigation revealed the presence of ETV6-ABL1. This finding renders necessary to determine DEK-CAN- and ETV6-ABL1-related features when using FKH1. This can be done as ETV6-ABL1 activity in FKH1 is responsive to imatinib. Nevertheless, we provided evidence that both SFK and mTOR activation in FKH1 are DEK-CAN-related features as they were activated also in other t(6;9) and DEK-CAN-positive models. The activation of STAT5 previously shown to be strong in t(6;9)-AML and activated by DEK-CAN is regulated in FKH1 by both DEK-CAN and ETV6-ABL1. In conclusion, FKH1 cells still represent a model for t(6;9)-AML and could serve as model for ETV6-ABL1-positive AML if the presence of these leukemia-inducing oncogenes is adequately considered.

Taken together, all our results provide clear evidence of novel and specific interdependencies between leukemia-inducing oncogenes and cancer signaling pathways which will influence the design of therapeutic strategies to better address the complexity of cancer signaling.

**Supplementary Information:**

The online version contains supplementary material available at 10.1007/s00277-022-04905-9.

## Introduction 

In acute myeloid leukemia (AML) the study of specific disease-defining translocations, such as t(15;17)-PML-RARα, t(8;21)-RUNX1-ETO or t(6;9)-DEK-CAN (also called DEK-NUP214) has allowed a better understanding of the molecular mechanisms of disease and recognition of the central role of aberrant transcription regulators in the pathogenesis of AML [1].

Three subtype specific translocations, namely t(15;17), t(11;17) and t(6;9), are notable for being associated with a small number of co-existing mutations, mainly in c-KIT or FLT3. In association with these translocations mutations in c-KIT or FLT3 are not essential either for induction or maintenance of the disease [2] and our unpublished data). This strongly suggests that the function of the aberrant translocation gene products may subsume the function of an otherwise compulsory combination of driver mutations, such as DNMT3A, TET2 and NPM1, found in normal karyotype AML [2].

AML with the t(6;9) is defined as a distinct entity by the WHO classification because of its particular biological and clinical features and unmet clinical needs [3]. In contrast to other AMLs, whose median age is around 66 years, most t(6;9)-AML patients are young, with a median age of 23–40 years [4, 5]. Complete remission (CR) rates do not exceed 50% and median survival after diagnosis is only about 1 year [6, 7]. Thus, the only current curative approach is haematopoietic stem cell transplantation (HSCT) in first CR [8]. These clinical facts show the urgent need of novel therapy approaches for t(6;9)-AML.

The t(6;9) represents the only structural chromosomal aberration present at diagnosis and encodes the DEK-CAN fusion protein [9]. Noticeably, the reciprocal CAN/DEK fusion transcript is not detectable making DEK-CAN the unique gene product of t(6;9) providing convincing evidence for the decisive role of DEK-CAN in leukemia initiation. While 30–75% of t(6;9)-AML patients harbour FLT3-ITD [10, 11], its presence accelerates the kinetics of relapse but seems not to be causally involved in leukemogenesis of t(6;9)-AML [8, 12]. This is consistent with our findings that DEK-CAN transforms immature haematopoietic stem and progenitor cells (HSPCs) in which the FLT3 promoter is not active [9]. Accordingly, t(6;9) remains an independent adverse risk feature, irrespective of the presence of FLT3-ITD [7, 13]. However, the mechanisms by which DEK-CAN transforms cells and mediates therapy-resistance have not been resolved.

It is known that the expression of the leukemogenic nuclear factor DEK-CAN activates the mTOR and STAT5 signaling [14, 15] strongly suggesting that activation of several signaling pathways contribute to the specific features by which DEK-CAN transforms cells in t(6;9)-AML. Therefore, we hypothesised the existence of a leukemogenic network of activated signaling pathways in t(6;9)-AML related to the expression of DEK-CAN.

For our investigation, we selected FKH1 cells as a model because it is the only t(6;9)-AML cell line available. The FKH1 cell line was described in 1998 as first permanent cell line with t(6;9) derived from the peripheral blood of a Japanese patient with t(6;9) AML. It was transformed from a Philadelphia chromosome (Ph)-negative myeloproliferative neoplasia (MPN). The FKH1 expressed the *dek-can* chimeric transcript and was factor-dependent as it grew only in the presence of 10 ng/ml recombinant human granulocyte colony-stimulating factor (G-CSF), with a doubling time of 54 h. Its capacity of multi-lineage myeloid differentiation with the presence of macrophages, basophils, eosinophils and neutrophils suggested that FKH1 cells originated from a pluripotent myeloid stem cell [16]. Although derived from a patient with previous Ph-negative MPN it has been considered useful since alternative models of the rare t(6;9)-AML were lacking. Developing further as a cell line, FKH1 cells lost their reported factor-dependency (DSMZ.com).

To investigate a network of activated signaling pathways in t(6;9)-AML we first performed phospho-proteomics on FKH1 cells bioinformatically elaborated by the Search Tool for the Retrieval of Interacting Genes (STRING) algorithm in order to evidence signaling pathways activated in t(6;9)-AML. The findings were then confirmed in other models of DEK-CAN induced leukemia.

## Results

### Constitutive ABL1 kinase activity in t(6;9)DEK-CAN-positive FKH1 cells

**Fig. 1:**
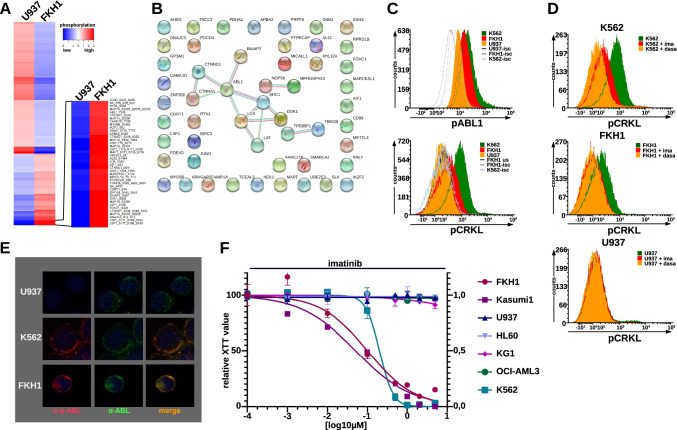
ABL1 kinase activity in t(6;9)-AML. **A** Phospho-proteomics analysis of FKH1 in comparison to U937 cells. The heatmap shows log2 transformed phosphorylation signals. It was generated by the gplot package (default settings) in RStudio and the indicated 40 most phosphorylated peptides were further analyzed. **B** STRING analysis of the 40 most phosphorylated peptides in FKH1 as compared to U937 cells. The type of supporting evidence (colour of connecting lines) was based on 4 criteria: (i) determined by experiments (purple); (ii) from curated databases (turquoise); iii. text-mining (yellow); and iv. protein homology (light blue). **C** pABL1 (Y412) and pCRKL (Y207) in FKH1 cells-IFC. U937–negative control; K562–positive control. **D** ABL1 substrate activation and its inhibition in FKH1 cells–IFC of pCRKL; ima (imatinib); dasa (dasatinib). The experiments shown in C and D were performed at least three times with similar results. **E** Immunofluorescence staining pattern of ABL1 (green fluorochrome) and pABL1 (red fluorochrome) in FKH1, K562-positive controls, and U937-negative control. All fluorochromes were merged electronically. **F** Response of FKH1 to imatinib as compared to other AML cell lines. Proliferation/cytotoxicity was measured by XTT after 5 days of exposure. The dose/response curve shows one representative of three independent experiments each performed in triplicates. K562-positive controls. dasa–dasatinib

We used quantitative phospho-proteomics to evidence proteins involved in aberrantly activated signaling pathways in t(6;9)-positive FKH1 cells in order to generate new hypotheses for further testing via biochemical and cell biological methods. Therefore, we compared FKH1 cells to DEK-CAN-negative U937. The total number of phosphopeptides reported in one run was 5569 (false discovery rate 5%). After filtering out the peptides matching to multiple proteins, 5534 phosphopeptides were kept for further analysis corresponding to 3851 unique peptide sequences that carried identical modifications. In this set, serine phosphorylation was detected on 3534 peptides (91.7%), threonine phosphorylation on 492 peptides (12.8%) and tyrosine phosphorylation on 69 peptides (1.8%). For each phosphopeptide, the position of the phosphorylation site(s) in the master protein was determined. The final intensity values were calculated by averaging the intensities across all phosphopeptides that contributed the same phosphorylation sites in the master protein. Of the resulting 3304 phosphoproteins, 1377 were differentially phosphorylated between FKH1 and U937 with a fold change difference of ≥ 1.5 up or down (Fig. 1A).

We focused on the 40 top upregulated phosphoproteins in FKH1 (Fig. 1A) and analyzed their potential physical and functional interactions by STRING [18]. Our STRING analysis was set at high confidence (interaction score of 0.7) and restricted to the first shell of interacting phosphoproteins (Fig. 1B). Given that the STRING analysis was based on up-regulated phosphoproteins in FKH1 cells, the outcome of this analysis (Fig. 1B) suggested the existence of a network that included ABL1 activity.

Constitutive ABL1 activity plays a crucial role in Philadelphia chromosome (Ph)-positive chronic myeloid leukemia (CML) and acute lymphoblastic leukemia (ALL) and AML as well as in Ph-negative MPN, Ph-like ALL and acute Tcell ALL (T-ALL) [19–22]. As constitutive ABL1 activity has never been reported in t(6;9)-AML, we wanted to investigate its activation status in t(6;9)-AML cells. We assessed ABL1 activity in t(6;9)-positive FKH1 cells, U937 (negative control), and Ph(BCR-ABL1)-positive K562 cells (positive control) by its autophosphorylation at Y412 and the phosphorylation of its substrate CRKL in IFC (intracellular flow cytometry) assays. In addition, we exposed the cells to the selective ABL1 kinase inhibitor imatinib and the dual ABL1/SFK inhibitor dasatinib. Here we show constitutive ABL1 activity in FKH1 both by ABL1 autophosphorylation and substrate phosphorylation of CRKL (Fig. 1C). This was confirmed by the fact that the ABL1 activity in FKH1 was responsive to inhibition by imatinib and dasatinib as it was in the positive control K562 (Fig. 1D).

Next, we studied the localisation of ABL1 activity in FKH1 and compared it to that of BCR-ABL1 in K562 cells. U937 cells were used as negative controls. Localisation was assessed by confocal laser scan microscopy (CLSM) using an anti p-ABL1-Y245 antibody (red fluorochrome) and an anti-total ABL1 antibody (green fluorochrome). The integrity of the nucleus was confirmed by DAPI staining. As shown in Fig. 1E, the same speckled cytoplasmic anti-p-ABL1-Y245 staining pattern was seen in both FKH1 and K562 cells, whereas no signal was seen in U937 cells. These findings suggested a role of ABL1 activity as survival signal in FKH1 cells similar to that played by BCR-ABL1 in K562.

To establish activated ABL1 as a survival signal, we investigated the effect of ABL1 kinase inhibition on growth of t(6;9)-positive cells. Hence, we exposed U937, OCI-AML3, KG1, HL60 together with FKH1 to increasing dosages of imatinib for 5 days. As positive controls, we used BCR-ABL1-positive K562 and Kasumi1 (positive for the c-KIT N822K). Growth arrest was assessed by an XTT cytotoxicity assay. The dosage dependent response of FKH1 cells to imatinib was comparable to that seen in Kasumi1 and K562 cells. All other cells did not respond to dosages of imatinib up to 2 µM (Fig. 1F).

These data provide evidence for ABL1 kinase activity being indispensable for the growth of t(6;9)-DEK-CAN-positive FKH1 cells.

### The ABL1 kinase activity in FKH1 is due to the presence of ETV6-ABL1 fusion

**Fig. 2:**
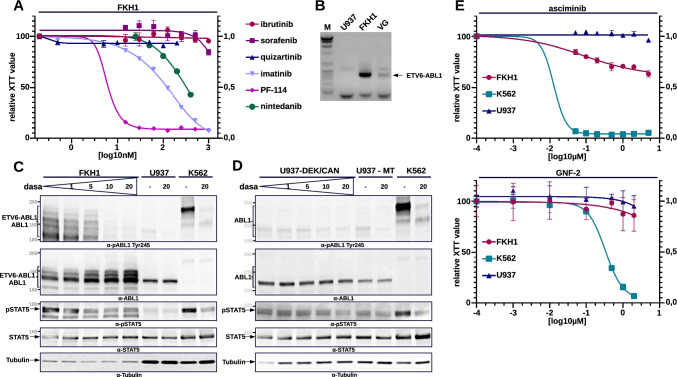
ABL1 activity in FKH1 and other DEK-CAN expressing cells. **A** Exposure of FKH1 to indicated TKIs-proliferation. The dose/response curves show one representative of three independent experiments each performed in triplicates. **B** RT-PCR on RNA obtained from FKH1, VG (ETV6-ABL1-positive ALL PD-LTC) and U937 (negative control) with the specific primers for the ETV6-ABL1 transcript (see “Material and methods” section). **C** ABL1 activation in FKH1 cells upon increasing concentrations of dasatinib–immunoblotting. The blots were stained with the indicated antibodies against phosphorylated (pABL1, pSTAT5) and total ABL1 and STAT5. STAT5 is a substrate of ABL1. **D** ABL1-STAT5 signaling in U937 cells expressing DEK-CAN upon inhibition by dasatinib–immunoblotting. The blots were stained with the indicated antibodies. **E** Efficacy of allosteric inhibition of ABL1 kinase in t(6;9)-positive AML cells by asciminib and GNF-2–proliferation/cytotoxicity was measured by XTT and dose response curve of one representative out of at least three experiments performed each in triplicate. K562–positive controls, U937–negative controls

In order to exclude receptor mediated ABL1 activation, we exposed FKH1 cells to a selection of inhibitors targeting the most important RTKs known in AML (see also supplementary Information). A response in FKH1 cells was seen only when the inhibitor was able to target ABL1 activity (Fig. 2A).

Although cytogenetically normal, the development of FKH1 cells in time led to a loss of factor-dependency [16] (DSMZ.com). In fact, we cultured these cells in absence of human G-CSF.

Recent preliminary data suggested the presence of a cryptic ETV6-ABL1 fusion in FKH1 [23]. For investigating the presence of an ETV6-ABL1 transcript in FKH1, we used primers routinely used for the detection of the ETV6-ABL1 fusion in diagnostics and as a marker of minimal residual disease (MRD) [24]. Here we performed a conventional reverse transcriptase (RT)-PCR assay. The patient-derived long-term culture (PD-LTC) VG was used a positive control. The PD-LTC VG was derived from a patient with T-ALL harbouring the t(9;12)-ETV6-ABL1 fusion [24]. As shown in Fig. 2B, FKH1 cells exhibited a strong ETV6-ABL1 signal at the same height of that in VG cells. No signal was seen in the U937 control cells. Therefore, we considered ETV6-ABL1 as responsible for the ABL1 activity in FKH1 cells and most likely the cause of the Ph-CML that developed into a t(6;9)-AML [16].

To confirm the presence of activated ETV6-ABL1 in FKH1 cells we performed immunoblotting on FKH1, U937 (negative control) and K562 (positive control). ABL1 activity was revealed by (i) its autophosphorylation detected by antibodies against pABL1-Y245 and total-ABL1; and (ii) its substrate phosphorylation of STAT5 detected by pSTAT5-Y694, and-total STAT5. As shown in Fig. 2C, a strong total and phospho-ABL1 signal was visible in FKH1 cells. It showed the pattern of a ladder that formed complexes up to ~ 230 KDa detected by the anti-pABL1 antibody and was susceptible to dasatinib inhibition as it was the substrate phosphorylation of STAT5 (Fig. 2C). Interestingly, the suppression of ABL1 activity led to an increase of total ETV6-ABL1 most likely as an expression of the significance of ABL1 activity for the survival of the cells (Fig. 2C). In control U937 cells, only a faint phosphorylated ABL1 band responsive to dasatinib was seen.

To further exclude that DEK-CAN is able to induce ABL1 activity, we studied U937 cells stably expressing DEK-CAN. As shown in Fig. 2E, we did not detect any DEK-CAN-induced ABL1-signal.

Although ETV6-ABL1-driven neoplasms are well responsive to ATP-competitive TKI such as imatinib, nilotinib and dasatinib, nothing is known to the best of our knowledge about the effects of allosteric inhibition on ETV6-ABL1. Differently to ATP-competitors, allosteric inhibitors inhibit the enzyme activity from outside the active center. The first in class allosteric inhibitor for ABL1-kinases is ABL001 (asciminib). It has been developed starting from GNF2 and 5 [25]. Therefore, we exposed FKH1 cells to both asciminib and GNF-2. Here we show that FKH1 did not exhibit a strong response either to asciminib or to GNF-2 (Fig. 2E). These findings strongly indicated that the ETV6-ABL1 activity in FKH1 was resistant to allosteric inhibition.

### STAT5 activity is regulated by ETV6-ABL1 and SFK in FKH1 cells

The presence of an ETV6-ABL1 activity complicates the use of FKH1 as a model for t(6;9)-AML and requires confirmation by other models independent of ETV6-ABL1. One characteristic of t(6;9)-AML is a strong STAT5 activity as revealed by proteomics on over 500 AML-patients [15]. STAT5 activation is dependent on the BCR-ABL1 kinase activity in Ph + leukemia cells and crucial for the maintenance of Ph + leukemia [26, 27]. Therefore, we wanted to understand how STAT5 activation was regulated in FKH1 cells as two potential regulators, ETV6-ABL1 and DEK-CAN, are present. That STAT5 activation was significant for the growth of t(6;9)-AML was shown by the response of FKH1 cells to AZD1208, a potent and selective inhibitor of PIM kinases, that are biological mediators of activated STAT5 [27]. In fact, FKH1 showed a dose-dependent growth reduction upon exposure to AZD1208 (Supplementary Fig. 1).

**Fig. 3:**
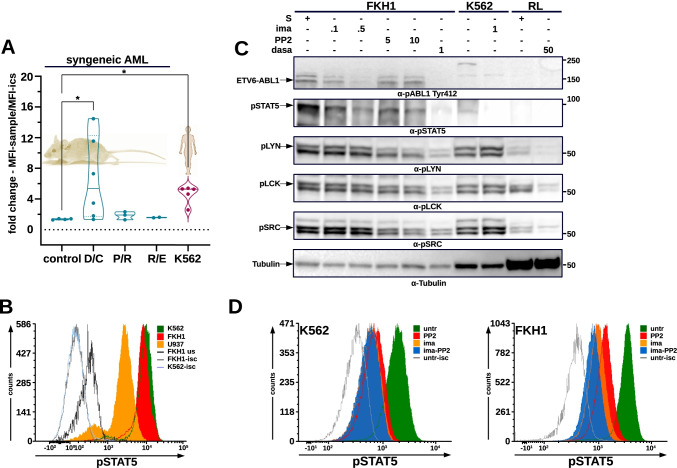
STAT5 regulation in FKH1 and other DEK-CAN expressing cells. **A** STAT5 activation in syngeneic AMLs–IFC analysis: fold change based on the MFI values in the sample relative to the MFI values of isotype controls. control–BM cells from healthy mice one year after transplantation with either empty vector or DEK-CAN transduced HSPCs; DC, PR and R1E–blast from syngeneic leukemias driven by DEK-CAN, PML-RARα or RUNX1-ETO, respectively. K562–positive control. **B**. IFC staining with anti pSTAT5 of FKH1. Green–K562; amber–U937; red–FKH1; isc–isotype control. **C** ABL1 and SFK–regulation of STAT5 activation. FKH1 exposed ON to PP2 (SFK), ima(tinib)(ABL1), dasa(tinib) (SFK/ABL1). Immunoblotting-membranes were stained with the indicated antibodies. K562, RL–positive controls. **D** IFC staining with anti pSTAT5 of FKH1 treated ON with indicated inhibitors. K562 (control). Green–untreated, amber–0.5 µM ima(tinib), red–10 µM PP2, blue–0.5 µM ima(tinib) + 10 µM PP2; isc–isotype control

First, we studied the capacity of DEK-CAN to phosphorylate STAT5. Therefore, we investigated STAT5 in primary syngeneic AMLs by comparing DEK-CAN-, PML-RARα- and RUNX1-ETO-induced leukemia. STAT5 activation was assessed by its phosphorylation detected by an anti-pSTAT5-Y694 antibody in IFC assays. Here, we show that STAT5 was phosphorylated not only in human t(6;9)-AML [15] to a higher level than in other AMLs but also in syngeneic DEK-CAN-positive leukemia (Fig. 3A). Next, we compared STAT5 phosphorylation in FKH1 with that of K562 and U937 by IFC. FKH1 cells exhibited phosphorylated STAT5 similar to that seen in K562 driven by BCR-ABL1 (Fig. 3B).

STAT5 activation can be triggered also by SFK and an involvement of activated members of SFK family in the regulation of STAT5 was already predicted by our phospho-proteomics and STRING analyses (Fig. 1B). Dasatinib is not a selective ABL1 inhibitor but a dual ABL1/SFK inhibitor and thus it inhibits not only ABL1-activity but also members of the SFK, including LYN, LCK and SRC, other known STAT5 regulators [28]. As we wanted to understand whether there is the possibility to functionally separate ETV6-ABL1 activity from that of DEK-CAN in this model, we investigated the role of SFK for the regulation of STAT5 in FKH1 cells. We used PP2 for the selective inhibition of SFK in comparison to that of ETV6-ABL1 by imatinib in order to unravel the individual roles of SFK and ABL1 for the regulation of STAT5. K562 treated with imatinib and RL lymphoma cells treated with dasatinib served as controls for ABL1- and SFK-activation, respectively. Here we show that PP2 inhibited all pLYN, pLCK and pSRC, but not pABL1, whereas imatinib inhibited efficiently ABL1 without any effect on SFKs (Fig. 3C). Interestingly, both PP2 and imatinib were able to reduce STAT5-phosphorylation in FKH1 cells indicating STAT5 being regulated by both ETV6-ABL1 and members of SFK in the FKH1 cell line.

Next, we studied the respective contribution of ABL1 and SFKs to the regulation of STAT5 by a sort of subtractive approach. We reasoned that if both, ETV6-ABL1 and SFKs were involved in the activation of STAT5 in FKH1 cells, the combined inhibition of ABL1 by imatinib and SFK by PP2 would have a stronger effect than the selective ABL1 inhibition alone. As a control we used BCR-ABL1-positive K562. Here we show that in FKH1 cells the combination of imatinib and PP2 had a stronger effect on the pSTAT5 signal than imatinib alone, whereas in K562 PP2 did not add to the effect of imatinib alone (Fig. 3D).

These findings provide evidence that in FKH1 STAT5 activation depends on both ETV6-ABL1 and SFK activation, whereas in K562 it depends on BCR-ABL1.

### Inhibition of SFK affects the growth of DEK-CAN-positive cells

**Fig. 4:**
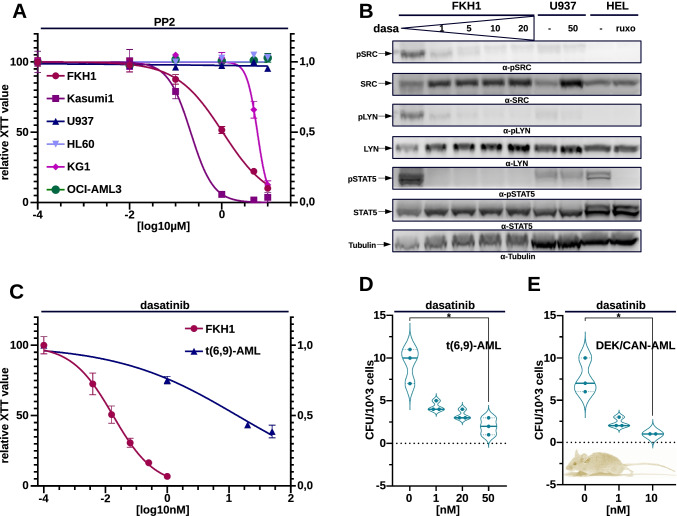
SRC family kinase (SFK) activation in t(6;9)-positive AML–regulation of STAT5 activation. **A** Proliferation of indicated cell lines upon ON treatment with PP2—the dose/response curves represent one of three independent experiments each performed in triplicates. **B** SFK inhibition in FKH1. ON exposure to dasa(tinib). The indicated antibodies detected phosphorylated and total SRC, LYN and STAT5. Tubulin–loading control. U937 and HEL cells ± ON treatment with ruxolitinib (ruxo)-controls. **C**–**E** SFK inhibition in t(6;9)-AML. **C** Proliferation of FKH1 and primary t(6;9)-AML blasts cells upon ON treatment with dasatinib. The dose/response curve shows one representative of three independent experiments each performed in triplicate. **D** CFU assay in semi-solid medium on human t(6;9)-AML blasts exposed to dasatinib—day 10. Colony numbers represent the mean of triplicates of one representative experiment out of two performed. **E** SFK inhibition in syngeneic DEK-CAN-AML–CFU-assay on syngeneic blasts. Colony numbers represent the mean of three (n = 3) experiments each performed in triplicates

In order to understand the significance of SFK activation for the phenotype of FKH1 cells, we wanted to answer the question of whether SFK inhibition by PP2 would interfere with the growth of these cells. We exposed FKH1 cells to the SFK inhibitor PP2 and showed that their growth assessed by XTT was inhibited in a dosage dependent manner. Only Kasumi1 cells were more sensitive than FKH1 (Fig. 4A).

TKIs used for the treatment of Ph + leukemia such as dasatinib are dual ABL1/SFK inhibitors that combine in one compound the inhibition of two kinases shown to be indispensable for the growth of FKH1 cells. Thus, we exposed FKH1 cells to increasing concentrations of dasatinib to investigate its effect on the SFKs SRC and LYN, as well as on STAT5. SFK and STAT5 activation was addressed by their autophosphorylation. As controls, we used U937 and HEL cells. HEL are dependent on JAK2-V617F, Thus, we inhibited the JAK2/STAT5 axis in these cells by ruxolitinib as a control. Dasatinib suppressed SFK LYN and SRC as well as the activation of STAT5 (Fig. 4B).

As nothing is known about the relationship between DEK-CAN and SFK we investigated the effects of dasatinib on the growth of t(6;9)-positive leukemia in three different models: FKH1 cells, t(6;9)-positive leukemic blasts and syngeneic DEK-CAN AML cells [15]. Growth was assessed by XTT and colony formation in semi-solid medium. Here we show that growth of FKH1 cells was inhibited by dasatinib at pM levels (Fig. 4C). This was confirmed by the effect of dasatinib on CFU formation of primary human t(6;9)-AML blasts as well as of syngeneic DEK-CAN-AML cells (Fig. 4D, E).

As DEK/CAN was not able to activate ABL1 kinase, we attributed the effects of dasatinib mainly to its ability to inhibit SFK members in these models. Therefore, we conclude that SFK activity contributes to the leukemic phenotype induced by DEK-CAN.

### mTOR signaling is a potential therapeutic target in t(6;9)-AML

**Fig. 5:**
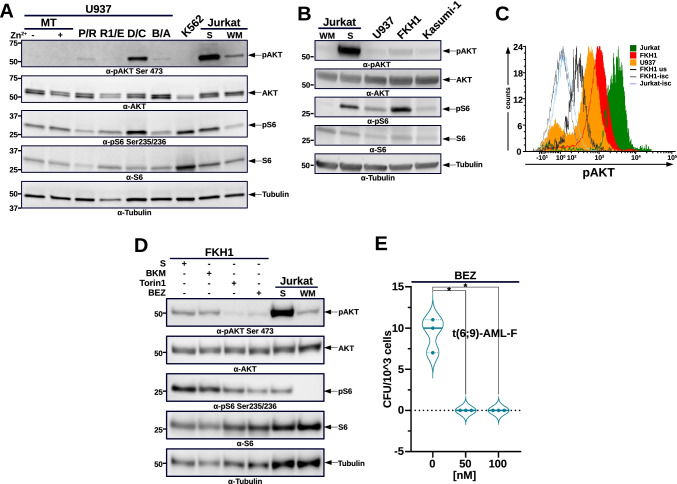
mTOR/AKT signaling in t(6;9)-AML. **A** Activation of mTOR/AKT axis in genetically modified U937. The indicated transgenes (P/R–PML-RARα; R1/E–RUNX1-ETO; D/C–DEK-CAN; B/A–BCR-ABL1) are expressed under the control of a Zn^2+^-inducible metallothionein (MT) promoter. U937 MT (empty vector transfected) ± Zn.^2+^. K562 and Jurkat–positive controls. S–solvent; WM–wortmannin. The blots were stained with the indicated antibodies. Tubulin–loading control. **B** mTOR signaling in patient-derived AML cell lines. U937, Jurkat–negative and positive controls, S–solvent; WM–wortmannin; FKH1–t(6;9)-DEK-CAN; Kasumi1–t(8;21)-RUNX1-ETO. **C** Detection of phosphorylated AKT (Ser473) in FKH1 cells by IFC. U937–negative control; Jurkat–positive control. Here is shown one representative of at least three independent experiments performed. **D** PI3K/AKT/mTOR inhibition in FKH1 cells. Jurkat–positive controls, S–solvent; WM–wortmannin. Inhibitors NVP-BKM123–PI3K/AKT (BKM); Torin1–mTOR; NVP-BEZ235–PI3K/mTOR (BEZ). All inhibitors were used at 50 nM ON. The blots were stained with the indicated antibodies. **E** mTOR inhibition in primary human t(6;9)-AML blasts by BEZ-CFU in semi-solid medium. The colony numbers represent the mean of two independent experiments

Another feature in t(6;9)-AML is the activation of mTOR signaling by DEK-CAN [14]. In order to further confirm mTOR activation being directly related to DEK-CAN expression, we extended the investigation to genetically modified U937 cells expressing either t(15;17)-PML-RARα (P/R), t(8;21)-RUNX1-ETO (R1/E), t(6;9)-DEK-CAN (D/C) or t(9;22)-BCR-ABL1 (B/A) (Fig. 5A). The U937 models excluded potential effects due to different genetic backgrounds. mTOR activation was assessed by anti pAKT-Ser473 and pS6 staining in immunoblotting and controlled by Jurkat cells in absence/presence of the TKI wortmannin (WM) [29]. Here we show that DEK-CAN was the strongest activator of mTOR signaling in U937 cells as compared to PML-RARα or BCR-ABL1 (Fig. 5A). As the mTOR activation seemed to be associated to DEK-CAN we investigated mTOR activation in FKH1 cells and showed that phosphorylated AKT and pS6 levels were higher in FKH1 cells than in other AML cells such as U937 or Kasumi1 cells (Fig. 5B). In order to quantify this activation we performed an anti-pAKT-Ser473 IFC on FKH1 in comparison to U937 (negative control) and Jurkat cells (positive control) that showed a 4–fivefold increase of AKT signaling in FKH1 with respect to U937 cells (Fig. 5C).

To confirm AKT phosphorylation via mTOR activation (mTORC2) and not via PI3K, we exposed FKH1 cells to three pharmacological inhibitors with distinct inhibitory properties: Torin1, a combined mTORC1/C2 inhibitor, NVP-BEZ235 (BEZ), a dual PI3K and mTORC1/C2 inhibitor, and NVP-BKM120 (BKM), a selective PI3K inhibitor. Only those compounds with mTOR inhibiting activity (Torin1 and BEZ) suppressed the activation of the AKT/S6K cascade in FKH1 cells (Fig. 5E).

Activation of mTOR signaling is central for cell survival, cell growth and proliferation [30]. To determine the degree of dependency on mTOR activation for growth, we studied the effects of the mTOR inhibitor BEZ on primary t(6;9)-AML blasts. Growth was assessed by CFU formation. BEZ inhibited CFU formation of primary t(6;9)-AML blasts (Fig. 5E). We exposed also FKH1 cells to BEZ and Torin1 and measured their effects on growth by an XTT assay. Both inhibitors arrested growth of FKH1 cells in a dosage dependent manner similar to Jurkat cells (Supplementary Fig. 2).

These data clearly showed that mTOR signaling is crucial for the survival of t(6;9)-AML.

### SFK/ABL1- and mTOR-inhibition is effective on t(6;9)-AML blasts

**Fig. 6:**
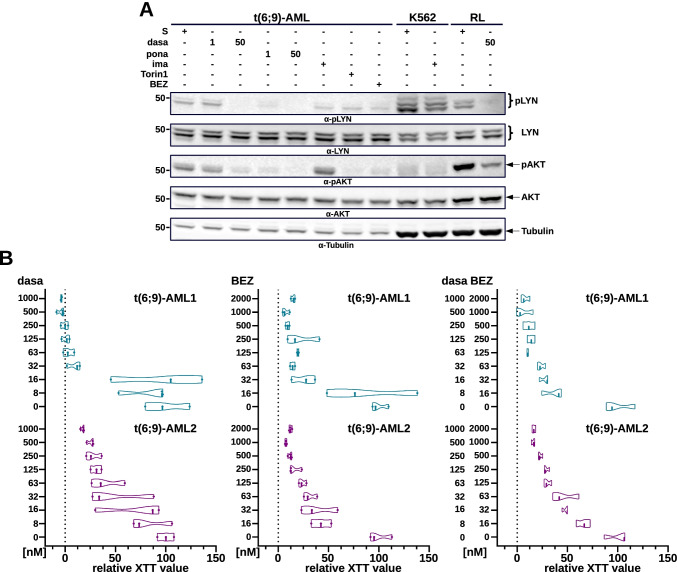
SFK/ABL1 and mTOR inhibition in t(6;9)-AML. **A** Interplay between ABL1-(ima), SFK-(dasa and pona) and mTOR- (BEZ, Torin1) inhibition in primary t(6;9)-blasts. The blots were stained with the indicated antibodies. **B** SFK/ABL1 and mTOR inhibition in human t(6;9)-AML blasts. The relative XTT values are from one representative experiment out of three each performed in triplicate

Recently, a direct relationship between SFK and mTOR signaling has been described [31]. In fact, dasatinib was able to inhibit mTOR signaling via the inhibition of LYN [32]. In order to confirm this relationship and the capacity of dasatinib to inhibit mTOR signaling we exposed primary t(6;9)-AML blasts to dasatinib, ponatinib, Torin1, and BEZ for studying the effects of these inhibitors on LYN and mTOR/AKT activation. Imatinib (1 µM) was used to exclude that the inhibition of ABL1 activity would have an effect on SFK or mTOR signaling. As shown in Fig. 6A, phosphorylation of both LYN and AKT was reduced upon exposure to dasatinib and ponatinib not only in the blasts but also in the RL cells used as positive control for LYN phosphorylation. On the other hand, Torin1 and BEZ only inhibited pAKT but not the phosphorylation of LYN (Fig. 6A), whereas imatinib did not show any effect.

The functional relationship between LYN and mTOR signaling raised the question whether this could be exploited for a therapeutic approach in order to improve treatment of patients with t(6;9)-AML. Therefore, we investigated the effects of mTOR- and SFK-inhibition alone and in combination on primary t(6;9)-AML blasts from two different patients. Once again, we used dasatinib as SFK inhibitor and BEZ as mTOR inhibitor. Both SFK and AKT/mTOR inhibitors alone strongly reduced growth, whereas the combination had only slight if any additive effect on the growth of t(6;9)-AML blasts from both patients (Fig. 6B). The lack of an additive or even a synergistic effect indicated a strong functional relationship between SFK and mTOR signaling and that the inhibition of one would not add to the inhibition of the other.

Taken together these data identify SFK and mTOR signaling as as closely related but valid only as individual nodes for therapeutic targeting in a network of activated signaling pathways characterising DEK-CAN-positive AML cells.

## Discussion

We provide evidence for an interplay of activated signaling pathways in t(6;9)-AML that are depending on the expression of the DEK-CAN. Our findings show that at least STAT5, mTOR, and SFKs are involved in this interplay of signaling pathways creating a network with nodes that represent targets for molecular therapy approaches [14, 15].

Until now, the main focus regarding aberrant kinase signaling in AML lied on receptor tyrosine kinases (RTKs), e.g. c-KIT or FLT3 and related signaling pathways [33]. This led to the combination of kinase inhibitors against these RTKs with conventional chemotherapy. This approach has improved the outcome of many high-risk AML patients [34]. In this study, we used only FLT3 negative models of t(6;9) AML as FLT3-ITD inhibitors do not improve the poor prognosis of t(6;9), and the only curative approach is currently HSCT in first CR [8]. The fact that the inhibition of most of the RTK relevant in haematopoiesis, FLT3 included, does not have any effect on the growth of t(6;9)-AML supports our hypothesis that the activation of STAT5, SFK and mTOR is autonomous. We could show that their activation is related directly to the expression of DEK-CAN or partially due to the ETV6-ABL1 fusion in FKH1 cells and not to the activation of an autocrine or paracrine mechanism mediated by RTKs (Supplementary Fig. 4). This supports a focus of therapy approaches on the effects of t(6;9)-DEK-CAN oncogene [7].

FKH1 cells have to be carefully evaluated as a model for t(6;9)-AML because of the presence of ETV6-ABL1. ETV6-ABL1 is the result of the t(9;12) that in most of the cases is cryptic. Its presence in FKH1 cells is most likely due to the fact they were derived from a t(6;9)-AML developed from a previous Ph- MPN [16]. The role of ETV6-ABL1 as an haematopoietic oncogene is well known. In fact, it represents one species of activated ABL1 with a central role in a number of leukemias such as a subset of Ph-like cALL, T-ALL and others together with that in Ph-MPN [35]. As both ETV6-ABL1 and t(6;9) are rare events in AML, FKH1 can represent a model for both genetic modifications in AML always if carefully used. In the case of ETV6-ABL1, imatinib eliminates the activated ABL1 kinase activity. However, one can think to use FKH1 as a model for ETV6-ABL1-AML and in fact we showed that these cells although normally sensitive to imatinib did not respond to allosteric inhibition of ABL1. This indicates that the myristoyl-binding pocket (MBP) of ABL1 in ETV6-ABL1 is not accessible either because of a different folding as compared to BCR-ABL1 or it is mutated in FKH1 cells. This has to be further investigated in order to understand whether asciminib can be used for the therapy of ETV6-ABL1 driven acute or chronic leukemia.

STAT5 activation is highly significant for the survival of t(6;9)-AMLs and therefore its regulation is critical. Interestingly, the axis JAK2/STAT5 seems to be interrupted as ruxolitinib did not show any effect on activated STAT5 in t(6;9)-AML (Supplementary Fig. 3). As DEK-CAN transforms rare immature stem cells, its role might be even more important, as STAT5 activation has been shown to be fundamental for stem cell maintenance in AML [36]. In addition, there seems to be a functional interplay between mTOR signaling and STAT5 in t(6;9)-AML, where the inhibition of mTOR led to a further activation of STAT5, similar to that seen in AML and T cells [37, 38].

It remains to be determined which individual contribution every single signaling pathway has on drug resistance, stemness and leukemic proliferation in t(6;9)-AML and thus to the poor prognosis of t(6;9)-AML patients. Accumulating evidence suggests that SFKs are activated in AML and contribute to stemness, cell survival and drug resistance [39–41] which is further corroborated by our finding of SFK activation in t(6;9)-AML.

Does DEK-CAN need co-factors such as ETV6-ABL1 for the induction and maintenance of AML? A high proportion of t(6;9) patients harbour FLT3-ITD [13], which activates its own signaling cascade [42], but targeting FLT3-ITD does not improve significantly the prognosis of t(6;9)-AML patients [7]. On the other hand it seems that in a t(6;9)-AML in absence of FLT3-ITD there is an activation of other signaling pathways such as RAS [2] although DEK-CAN alone is able to induce AML in vivo.

Here, we present data that will modify current concepts of pathogenesis of AML. Our findings suggest that at least for certain subgroups of AML some of the mutations, such as DEK-CAN or PML-RARα are able by themselves to aberrantly activate signaling pathways essential for the survival and the biology of the respective AML cell. Interestingly, there is no need for these factors to be mutated in order to be constitutively activated. This provides further evidence for the need of analyses such as phospho-proteomics during the development of targeted therapies to understand not only the activation status of the entire signaling structure within a tumor cell but also the effects of an inhibitor on this network and potential positive feed-back effects that might lead to drug resistance.

Taken together our here presented data will modify the design of molecular therapy by suggesting the need for a broader approach aimed to include not only one but more signaling pathways, which eventually requires a well-studied combination of inhibitors.

## Materials and methods

### Compounds

All inhibitors used in this study were purchased from Selleckchem (www.selleckchem.com). For all compounds 1000 × stock solutions were obtained by dissolving the compounds in dimethyl sulfoxide (DMSO)(Sigma) then further diluted to working concentrations in the corresponding cell culture medium or semi-solid medium prior to use.

### Cell lines and cell culture

All cell lines (FKH1, HEL, HL60, JURKAT, K562, Kasumi1, KG1, OCI-AML3, RL, U937,) were obtained from the German Collection of Microorganisms and Cell Cultures (DSMZ), Braunschweig, Germany, and were kept in RPMI-1640 medium supplemented with 10% FCS except for FKH1 and Kasumi-1 cells which were maintained in 20% FCS. The U937 clones were created either by M Ruthardt’s laboratory or a kind gift from P.G. Pelicci (IEO, Milan): MTB45 (empty vector-control), PR9 (expressing PML-RARa), RUNX-1/ETO (expressing HA-RUNX-1/ETO), BCR/ABL or DEK/CAN (expressing HA-DEK/CAN)(10). All these cells expressed their respective transgene under the control of MT-1 promoter inducible by 100 µM ZnSO4 (Zn^2+^) [17].

### Syngeneic leukemia

Different types of primary syngeneic leukemic cells stored in liquid N_2_ and thawed for further analysis were obtained from leukemic mice injected with primary murine HSPCs transduced with leukemia-inducing oncogenes (PML-RARa, RUNX-1/ETO, BCR/ABL, and DEK/CAN) as described elsewhere [9, 15]. As control, we used cells from healthy recipients injected with empty vector transduced HSPC [9, 15].

### Patient samples

Primary human AML samples were obtained with informed consent and used in agreement with the Declaration of Helsinki upon the approval of the local ethic committees of the Goethe University Frankfurt (approval number 329–10). Samples were maintained in X-Vivo10 medium (Lonza) supplemented with 10% FCS (Hyclone/Perbio Science), with the addition of 20 ng/ml hIL-3, 50 ng/ml hSCF, 25 ng/ml hTPO and 50 ng/ml hFLT3-ligand (Miltenyi, Bergisch-Gladbach, Germany).

### Immunodetection

#### Immunoblotting

Immunoblot analyses were performed according to widely established protocols. The following kits and antibodies were used: mTor Substrates Antibody Sampler Kit^©^, Phospho-Akt Pathway Antibody Sampler Kit^©^, anti-pSTAT5-Y694, anti-pABL1-Y245 and Y412, anti-pSRC-Y416, anti-SRC, and anti-pLCK-Y505 (all from Cell Signaling). Anti-ABL1, anti-STAT5 (Santa Cruz Biotechnology); anti-pLYN-Y396 (Abcam), anti-LYN (BD Biosciences), anti-α-Tubulin (Lab Vision). Blocking and antibody incubation were performed in 5% low-fat dry milk (Carl Roth). Washing was performed in Tris-buffered saline containing 0.1% Tween20 (TBS-T) followed by incubations either with secondary Ab coupled with horseradish-peroxidase for staining with enhanced chemiluminiscence substrate or the IRDye R^©^ 800 and 680 goat anti rabbit or anti mouse ABs (LI-COR Biosciences). Blots were “stripped” using RestoreWestern blot Stripping Buffer^©^ (Perbio Science). Imaging and elaboration were performed with the LI-COR Odyssey Fc system (LI-COR Biosciences).

#### Intracellular flow cytometry (IFC)

5 × 10^5^ cells/sample were fixed with Cytofix buffer (BD Biosciences) according to the manufacturer’s protocol. Permeabilisation was performed with ice-cold 90% methanol for 30 min. Cells were then incubated with the primary antibody pSTAT5-Y694-AlexaFluor 647 (BD Biosciences), pAKT-S473-PE-Vio770, pCRKL-Y207-APC or IgG control for 40 min at RT (Miltenyi). For ABL1 staining, the cells were incubated with the non-labelled pABL1-Y245 (Cell Signaling Technologies) followed by secondary goat anti-rabbit IgG AlexaFluor-488 Fab2 (Cell Signaling Technologies). Washing was performed with PBS containing 1% FCS and 0.1% sodium azide. Fluorescence was measured immediately after staining on a FACS Fortessa (BD Biosciences).

#### Confocal laser scan microscopy (CLSM)

Cells were cultured over-night on poly-D lysine covered chamber slides (Corning), washed with TBS (10 mmol/L Tris–HCl pH 8, 150 mmol/L NaCl), fixed in 4% paraformaldehyde (AppliChem) for 15 min, and permeabilised with 0.1% Triton-X in TBS. Blocking was performed in 3% (w/v) BSA (Sigma) and 0.1% Tween20 in TBS for 60 min. Cells were incubated with polyclonal rabbit anti–pABL1 (Y245)(Cell Signaling) or monoclonal anti–c-ABL (Santa Cruz) followed by. Alexa Fluor 647-conjugated goat anti-rabbit or anti-mouse AlexaFluor-488 Fab2 Ig antibodies (Life Technologies). Nucleus staining was obtained using DAPI (Life Technologies). The slides were mounted with Moviol (Sigma). Images were acquired by a Leica TCS-SP5 confocal microscope (Leica Microsystems) under identical conditions for pinhole opening, laser power, photomultiplier tension and layer number. Identical parameters were applied for all samples during data elaboration by Imaris (Oxford Science). Final picture elaboration (cutting, contrast) was performed with open access GIMP-software.

#### Proliferation/cytotoxicity

Proliferation was assessed by using the XTT proliferation kit according to the manufacturer’s instructions (Roche).

#### Phospho-proteomics

For phosphoproteomic analysis the FKH1 and the U937 cells were splitted at a density of 250,000 cells/ml and allowed to rest overnight before lysis.

#### Tandem mass tag (TMT) labeling and phosphopeptide enrichment

Aliquots of 100 µg of up to 10 samples per experiment were digested with trypsin (2.5 µg trypsin per 100 µg protein; 37 °C, overnight), labeled with TMT ten plex reagents according to the manufacturer’s protocol (Thermo Fisher Scientific) and the labeled samples pooled. This pooled sample was then desalted using a SepPak cartridge (Waters) and subjected to TiO2-based phosphopeptide enrichment according to the manufacturer’s instructions (Pierce). The phospho-enriched sample was evaporated to dryness and then resuspended in 1% formic acid prior to analysis by nano-LC MSMS using an Orbitrap Fusion Tribrid mass spectrometer (Thermo Fisher Scientific).

#### Nano-LC mass spectrometry

The TMT-labelled phospho-enriched sample was fractionated using an Ultimate 3000 nano-LC system in line with an Orbitrap Fusion Tribrid mass spectrometer (Thermo Fisher Scientific). In brief, peptides in 1% (vol/vol) formic acid were injected onto an Acclaim PepMap C18 nano-trap column (Thermo Scientific). After washing with 0.5% (vol/vol) acetonitrile 0.1% (vol/vol) formic acid peptides were resolved on a 250 mm × 75 μm Acclaim PepMap C18 reverse phase analytical column (Thermo Fisher Scientific) over a 150-min organic gradient, using 6 gradient segments (5–9% solvent B over 2 min, 9–25% B over 94 min, 25–60%B over 23 min, 60–90%B over 5 min, held at 90%B for 5 min and then reduced to 1%B over 2 min.) with a flow rate of 300 nl min^−1^. Solvent A was 0.1% formic acid and solvent B was aqueous 80% acetonitrile in 0.1% formic acid. Peptides were ionised by nano-electrospray ionisation at 2.0 kV using a stainless-steel emitter with an internal diameter of 30 μm and a capillary temperature of 275 °C. All spectra were acquired using an Orbitrap Fusion Tribrid mass spectrometer controlled by Xcalibur 2.0 software (Thermo Fisher Scientific) and operated in data-dependent acquisition mode using an SPS-MS3 workflow. FTMS1 spectra were collected at a resolution of 120,000, with an automatic gain control (AGC) target of 200,000 and a max injection time of 50 ms. The TopN most intense ions were selected for MS/MS. Precursors were filtered according to charge state (to include charge states 2–7) and with monoisotopic precursor selection. Previously interrogated precursors were excluded using a dynamic window (40 s ± 10 ppm). The MS2 precursors were isolated with a quadrupole mass filter set to a width of 1.2 m/z. ITMS2 spectra were collected with an AGC target of 5000, max injection time of 120 ms and CID collision energy of 35%. For FTMS3 analysis, the Orbitrap was operated at 60 000 resolution with an AGC target of 50,000 and a max injection time of 120 ms. Precursors were fragmented by high energy collision dissociation (HCD) at a normalised collision energy of 55% to ensure maximal TMT reporter ion yield. Synchronous Precursor Selection (SPS) was enabled to include up to 5 MS2 fragment ions in the FTMS3 scan.

#### Data analysis

The raw data files were processed and quantified using Proteome Discoverer software v2.1 (Thermo Scientific) and searched against the Uniprot Human database (134,169 sequences) using the SEQUEST algorithm. Peptide precursor mass tolerance was set at 10 ppm, and MS/MS tolerance was set at 0.6 Da. Search criteria included oxidation of methionine (+ 15.9949) and phosphorylation of serine, threonine and tyrosine (+ 79.966) as variable modifications and carbamidomethylation of cysteine (+ 57.0214) and the addition of the TMT mass tag (+ 229.163) to peptide N-termini and lysine as fixed modifications. Searches were performed with full tryptic digestion and a maximum of 1 missed cleavage was allowed. The reverse database search option was enabled and all peptide data was filtered to satisfy false discovery rate (FDR) of 5%. All additional bioinformatic elaboration was performed in Rstudio.

The mass spectrometry proteomics data have been deposited to the ProteomeXchange Consortium via the PRIDE partner repository with the dataset identifier PXD031429.

### Search Tool for the Retrieval of Interacting Genes (STRING)

For the analysis, the STRING package provided on the uniprot website was used (www.uniprot.org). STRING acts as a meta-database that examines the potential of both physical and functional protein–protein interactions. The outcome is based on information from numerous sources, including experimental repositories, computational prediction methods and public text collections.

### Colony forming unit assays (CFU) on syngeneic leukemia cells and primary cell culture of human t(6;9)-AML blasts

For a CFU on murine leukemic cells freshly thawed BM cells from DEK/CAN-positive leukemic mice and healthy empty vector transduced control transplanted mice were plated in semi-solid medium supplemented with mIL-3 (20 ng/mL), mIL-6 (20 ng/mL) and mSCF (100 ng/mL) (Stem-Cell Technologies). On day 10 after plating, the number of colonies was determined. Freshly thawed bone marrow cells derived from t(6;9)-positive AML patients were cultured in semi-solid medium (MethoCult™ H4434, Stem-Cell Technologies) in the presence of the indicated compounds for 14 days, and the colony number was determined in comparison to the untreated samples.

### Statistics

The statistical analysis of the proliferation inhibition results was performed using GraphPad Prism9. The dose–response curves were fitted using the nonlinear regression model and the log(inhibitor) vs normalised response-variable slope function. The comparison between groups was performed using one-way analysis of variance and the non-parametric Kruskal–Wallis test. The multiple comparisons between conditions were corrected by controlling the false-discovery rate using the Benjamin-Hochberg method.

## Supplementary Information

Below is the link to the electronic supplementary material.

Supplementary file1 (PDF 270 KB)
