# Supplementary figures and images for: Activation of signaling pathways in models of t(6;9)-acute myeloid leukemia
Source: Ann Hematol. 2022 Aug 8;101(10):2179–93. doi: 10.1007/s00277-022-04905-9 (PMC9463248; doi:10.1007/s00277-022-04905-9)

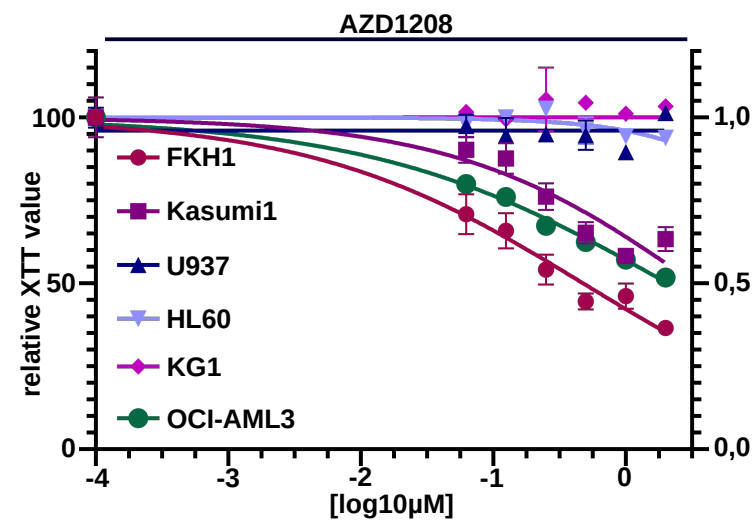

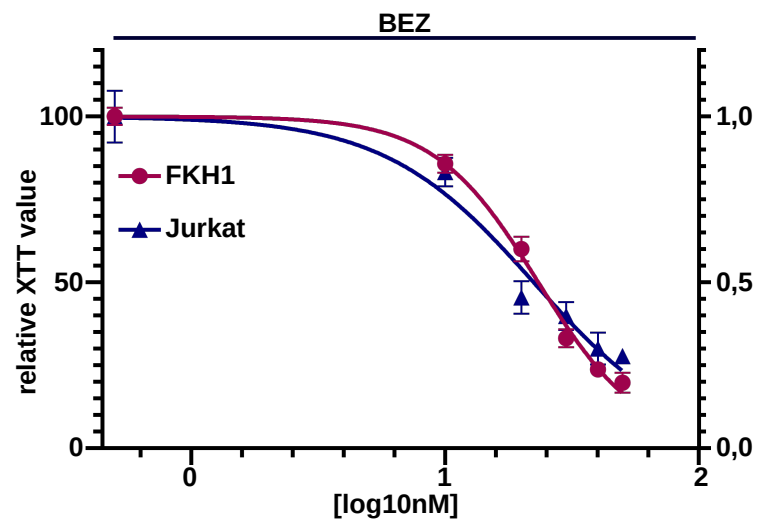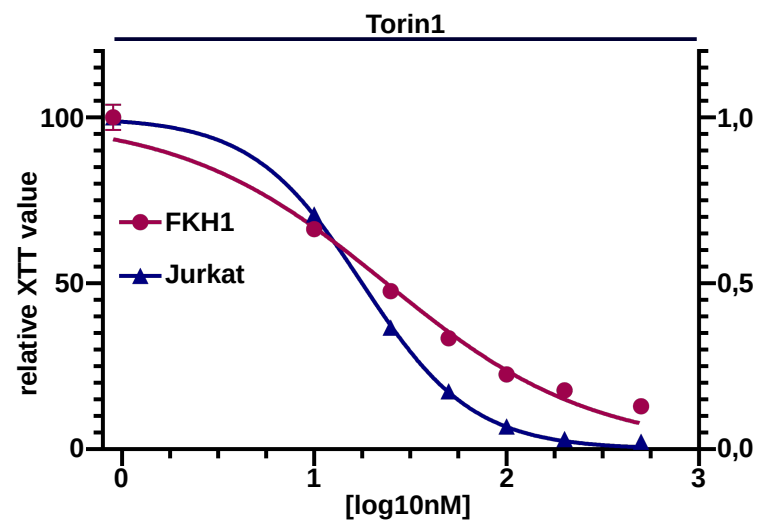

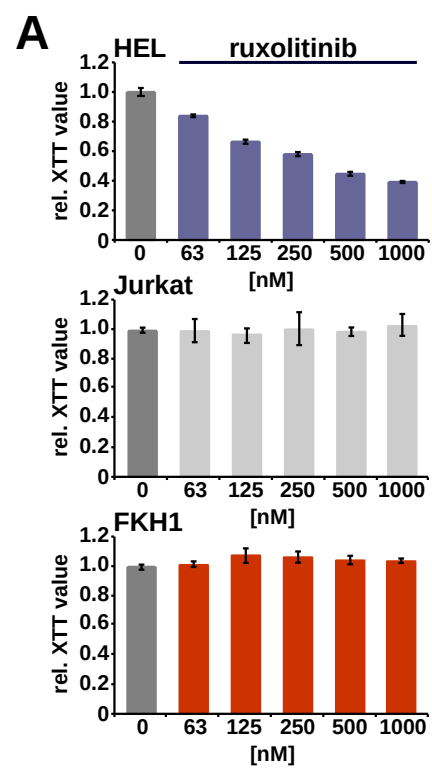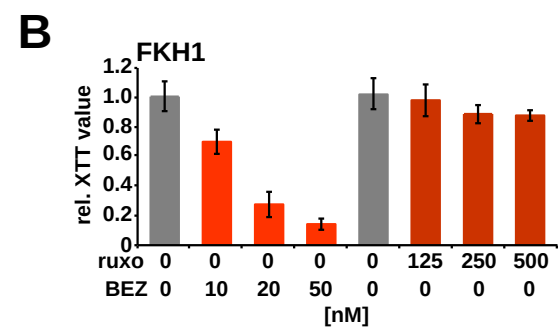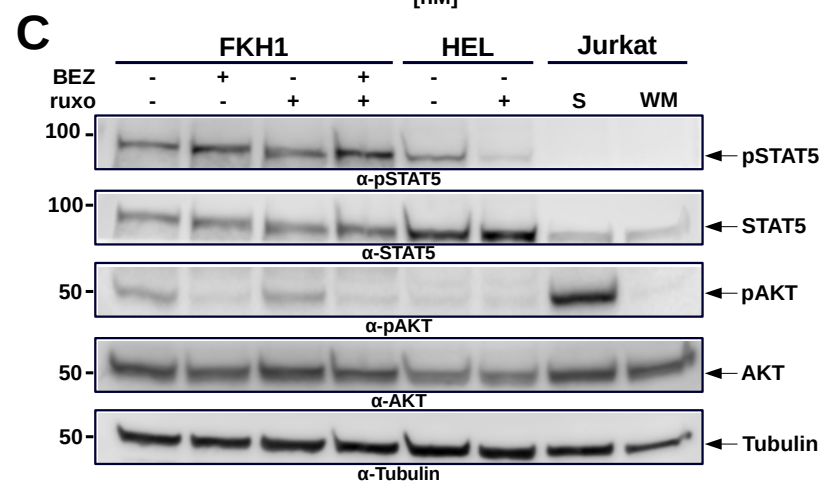

ETV6-ABL1

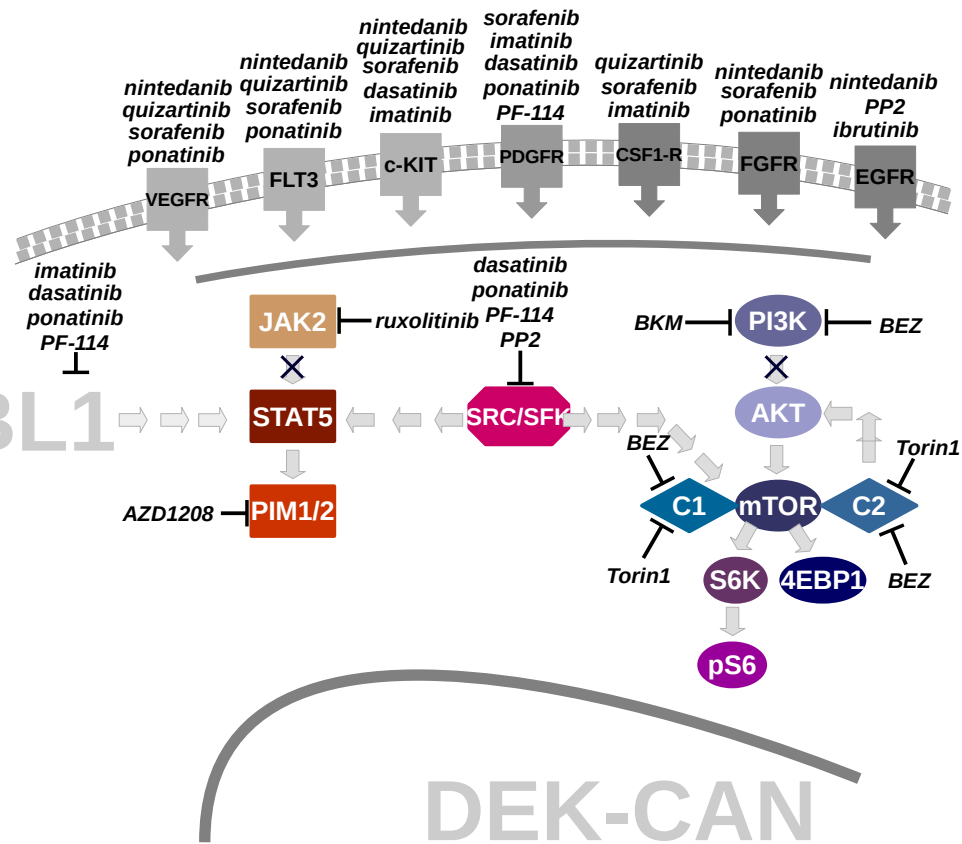

Supplement: Supplementary file 1 — Supplementary file1 (PDF 270 KB) [file 277_2022_4905_MOESM1_ESM.pdf]
